# Spatial-Temporal Patterns of Air Pollutant Emissions From Landing and Take-Off Cycles in the Yangtze River Delta of China During the COVID-19 Outbreak

**DOI:** 10.3389/fpubh.2021.673666

**Published:** 2021-09-07

**Authors:** Danwen Bao, Shijia Tian, Ziqian Zhang, Hao Cheng, Ting Zhu, Nicholas Carpeggiani

**Affiliations:** ^1^College of Civil Aviation, Nanjing University of Aeronautics and Astronautics, Nanjing, China; ^2^School of Engineering, Royal Melbourne Institute of Technology, Melbourne, VIC, Australia

**Keywords:** aircraft emission, COVID-19, LTO cycles, civil aviation, Yangtze River Delta (YRD)

## Abstract

The global aviation industry has been experiencing catastrophic disruption since the beginning of 2020 due to the unprecedented impact of the COVID-19 pandemic on air traffic. Although the decline in regular commercial air travel has caused tremendous economic loss to aviation stakeholders, it has also led to the reduction in the amount of recorded air pollutants. Most of the aircraft emissions are released during the cruise phase of flight, however they have relatively small impact on humans due to the fact that those emissions are released directly into the upper troposphere and lower stratosphere. Therefore, the scope of this study is to investigate the ground-level aircraft emissions from landing and take-off (LTO) cycles, as they have a greater influence on the ambient environment of the airports in a specific region. In this paper, we study the variation of typical air pollutant concentrations (i.e., HC, CO, and NO_x_) from the LTO cycles during the outbreak of COVID-19 pandemic in both temporal and spatial scales. These ground-level emissions are estimated for the 22 airports in the Yangtze River Delta, China. The results indicate that the variation pattern of the three air pollutants were significantly influenced by the dramatic onset of the COVID-19 pandemic, as well as the pertinent policies to suppress the spread of the virus. The results also reveal non-uniform distribution of the emission quantified at different airports. It is noticeable that the emission quantity generally declined from the east coast to the central and western part of the research region. Furthermore, discrepancies in the target markets also create disparities in the variation pattern of the emissions at different airports under the context of COVID-19.

## Introduction

First appearing in December of 2019, the coronavirus disease (COVID-19) has become a global health crisis. As of February 17, 2021, there have been more than 100 million confirmed cases of COVID-19, including 2.4 million deaths, worldwide (1). The virus is highly contagious and can be easily transmitted when in close contact with an infected person. Evidence shows that the virus is primarily to spread through respiratory droplets, which usually come from coughing and sneezing of the infected people, regardless of whether or not they are symptomatic (2). Due to this transmission pattern, national and local governments of China have imposed stringent restrictions on the movement of people and vehicles in an attempt to suppress the spread of COVID-19 after the initial outbreak in Wuhan City. The adopted policies include city lockdown, travel restrictions and stay-at-home requests (3).

As a result, the civil aviation sector has become more financially exposed than ever. Aviation experts suggest that the increasing passengers' concerns for their personal health have significantly decreased their willingness to fly for both business and pleasure (4). Evidence shows that passengers are at higher risks getting infected if there is a confirmed case within two passenger rows in an aircraft in spite of the fact of highly-efficient filter systems onboard aircraft (5). Thus, even though airlines and airports around the world have adopted various hygiene measures to minimize the risks of getting infected, the lack of public confidence in the air transportation system will maintain a substantial challenge for commercial aviation sector for the near future (4).

Although the contraction in regular commercial flight operations has caused tremendous financial loss to associated aviation stakeholders, positive effects on the environment due to the decline in aviation activities cannot be overlooked. According to Kang et al. (6), direct emissions from the aircraft engines sharply fell at a rate and scale that never observed before. Given the fact that the aviation activities make substantial contribution to the air pollutant emissions, it is anticipated that the air quality can be therefore improved under the context of COVID-19 (7).

Aircraft pollutant emissions have been of concern since the emergence of commercial aviation (8). According to the World Wildlife Fund (9), if the entire aviation sector were a nation, it would rank top 10 among the carbon-polluting countries on the planet. Air travel is also the activity with the highest emissions per individual traveler. Research shows that a passenger flying from New York to London round-trip generates more emissions than the average amount over the life-course of an ordinary person in Paraguay (9). Unlike the emissions resulting from the ground vehicles, aircraft emissions are unusual in that a significant proportion is emitted at high altitude. Subsequently, most of the aircraft emissions are released directly into the upper troposphere and lower stratosphere during the cruise phase, thus they have relatively small impact on humans (8). However, there are still ~25% of the emissions produced near the ground during the landing and take-off (LTO) cycles, which can directly impact the local air quality and the health of people living in the vicinity of the airport (10).

These aforementioned ground-level emissions have aroused increasing public concern in the recent years as they can have great influence on the ambient surrounding environment of the airports. The changing situation of the COVID-19 epidemic provides a unique opportunity to assess the variation pattern of the emissions at the airports over different geographical locations. Therefore, this paper studies the variation of some typical air pollutant concentrations (HC, CO, and NO_x_) from the LTO cycles within the context of COVID-19 pandemic. Those ground-level emissions are primarily estimated for the 22 airports in the Yangtze River Delta in east-center of China. The measurements taken were made over the entirety of 2020. Time series analysis and spatial distribution analysis are conducted respectively to investigate the variation pattern of HC, CO, and NO_x_ from the LTO cycles in both temporal and spatial scales. The results of the research may be important for the air quality management in the post period of the pandemic.

## Materials and Methods

### Research Region

**Figure 1:**
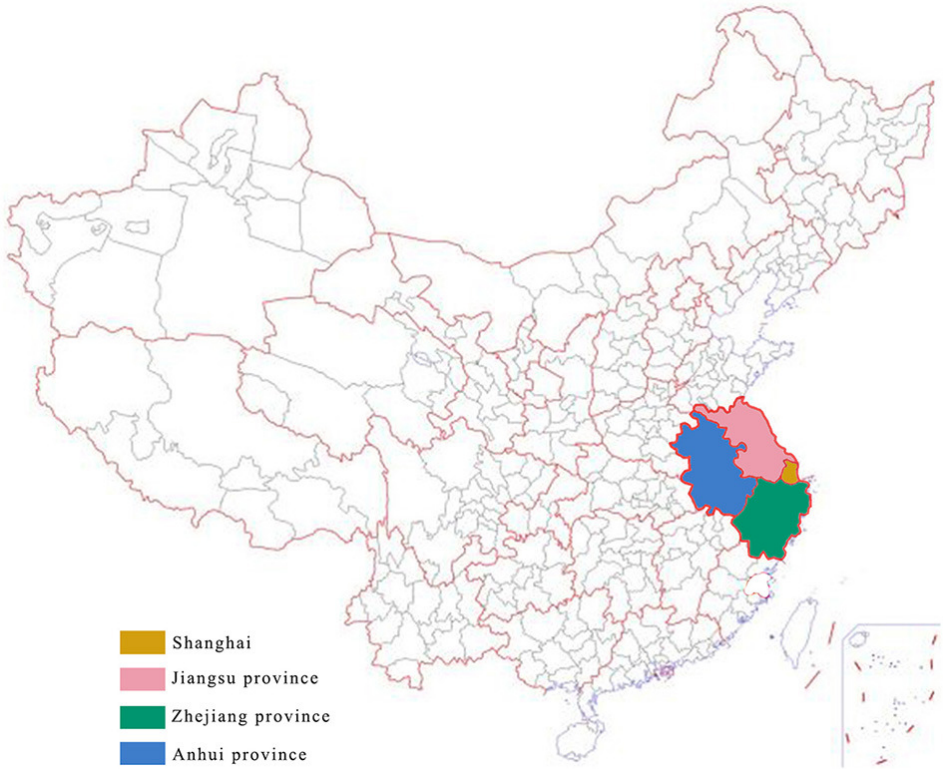
The location of Yangtze River Delta in China and the relative locations of the studied regions (i.e., Shanghai city, Jiangsu province, Zhejiang province, and Anhui province).

The research region of this paper is the Yangtze River Delta surrounding Shanghai, which is one of the four direct-administered municipalities of China. The research region is located in the eastern of China (Figure 1). It is one of the three most important economic regions of China. The other two regions are the Bohai Bay region near Beijing and the Pearl River Delta closed to Hong Kong. Airports in the Yangtze River Delta have developed rapidly with the growth of the city groups in the region. Currently, the Yangtze River Delta is the region with highest airport density in China, and the service radius of an airport often covers adjacent airports. There are 22 civil airports in the region. They are 2 airports in Shanghai (Shanghai Pudong Airport-PVG and Shanghai Hongqiao Airport-SHA), 9 airports in Jiangsu province (Nanjing Lukou Airport-NKG, Sunan Shuofang Airport-WUX, Changzhou Benniu Airport-CZX, Nantong Xingdong Airport-NTG, Lianyungang Baitabu Airport-LYG, Yancheng Nanyang Airport-YNZ, Xuzhou Guanyin Airport-XUZ, Huaian Lianshui Airport-HIA, Yangzhou Taizhou Airport-YTY), 6 airports in Zhejiang province (Hangzhou Xiaoshan Airport-HGH, Yiwu Airport-YIW, Ningbo Lishe Airport-NGB, Wenzhou Yongqiang Airport-WNZ, Zhoushan Putuoshan Airport-HSN, Taizhou Luqiao Airport HYN), and 5 Airports in Anhui Province (Hefei Xinqiao Airport-HFE, Huangshan Tunxi Airport-TXN, Anqing Tianzhushan Airport-AQG, Fuyang Xiguan Airport-FUG, Chizhou Jiuhuashan Airport-JUH).

### Data Sources

#### Flight Schedule Databank

Founded in Mainland China, VariFlight Technology Co. Ltd. is an international technology company that specializing in providing data service in the domain of civil aviation. One of the primary products of the company is flight status data that includes flight schedules, historical and live flight status and information on airports and aircrafts. VariFlight reached 100% coverage of domestic flights in 2009 and covered 94% of the global commercial flight data by 2017 (11).

With a close collaboration with VariFlight, the civil aviation college of Nanjing University of Aeronautics and Astronautics (NUAA) has built its own databank to store the information of all flight schedules of 22 airports in the Yangtze River Delta. Each record of the flight contains the following information: flight number, operating airline, the type of the aircraft, origin airport, destination airport, and flight status. The flight schedule of the 22 airports in the Yangtze River Delta from 1 January 2020 to 31 December 2020 were obtained from this databank. From the records of flights, the number of daily LTO cycle and aircraft type can be collected. All records have been thoroughly checked to avoid calculation mistakes (e.g., multiple times of calculation).

#### ICAO Engine Emission Databank

The International Civil Aviation Organization (ICAO) Aircraft Engine Emissions Databank provides information on the exhaust emissions of specific aircraft engines. For the operation of each type of aircraft engine in one intact LTO cycle, the databank contains the power setting (%), the time spent (minutes), fuel flow (kg/s) and the emission indices of the HC, NO_x_, and CO for each operation mode (i.e., idle, approach, climb-out, and take-off). All the measured data is in accordance with the procedures and recommended standards in ICAO Annex 16, Volume I. An example of the information for engine Trent 772 is shown in Table 1.

**Table 1:** The measured data for Trent 772.

**Mode**	**Power setting**	**Time (min)**	**Fuel flow (kg/s)**	**HC** **(g/kg)**	**CO** **(g/kg)**	**NOx** **(g/kg)**
Take-off	100	0.7	3.150	0.33	0.18	43.60
Climb- out	85	2.2	2.580	0.35	0.15	32.66
Approach	30	4.0	0.840	0.81	0.78	10.68
Idle	7	26.0	0.270	0.97	9.38	5.74

#### Other Resources

It is of great significance to identify the combination of engine types for a specific aircraft. The combination of aircraft and engine were obtained from the official websites and reports of aircraft manufacturers, the websites of air carriers and abundant academic literatures. For the reason that the same aircraft type may install different categories of engines even in the same air carrier, we select the most used engine type for each type of the aircraft (Table 2). The number of engines for each type of the aircraft can be acquired from the official websites of aircraft manufacturers.

**Table 2:** The combination of Aircraft/Engine for calculation.

**Aircraft type**	**Engine type**	**Aircraft type**	**Engine type**
A310	CF6-80A	B736/B737	CFM56-7B22
A319	CFM56-5B7-P	B738	CFM56-7B27
A320	V2527-A5	B739	CFM56-7B26
A321	CFM56-5B3/3	B744/B747	PW4056
A330/A332/A333	Trent 772	B767	CF6-80C2
A340/A343	CFM56-5C	B772/B777	GE90-77B
A346	Trent556	B773	GE90-94B
A350	Trent XWB-75	B787/B788/B789	GEnx-1B
A380/A388	Trent 970-84	E190	CF34-10E
B733	CFM56-3-BI		

### Research Methods

To assess the impacts of COVID-19 pandemic on air pollutant emissions from LTO cycles in the Yangtze River Delta, the emissions quantities of HC, CO, and NO_x_ are firstly calculated. Then, the research is carried out by comparing the variations of the 3 aircraft pollutants in year 2020 in both temporal and spatial scales.

#### Aircraft Emission Calculation Model

The principle emissions of aircraft engines include gases like carbon dioxide (CO_2_) carbon monoxide (CO), unburned hydrocarbons (HC) like methane (CH_4_), oxides of nitrogen (NO_x_), sulfur oxides (SO_x_) and particles like PM_2.5_ and PM_10_ (12). In Annex 16 published by ICAO, three main jet engine emissions (i.e., HC, NO_x_, and CO) were particularly addressed due to their influence on the environment and public health. Therefore, the research scope for the emissions in this paper is also the three gaseous emissions: HC, NO_x_, and CO.

In order to systematically measure and control the aircraft emissions, ICAO has defined reference emissions from LTO cycle (8). A typical LTO cycle consists of four modes of engine operation, i.e., idle, approach, climb-out and take-off. Each of the operation mode corresponds with specific power settings of the engines and typical operational time (13). To calculate the emissions of HC, NO_x_, and CO, we follow the method recommended by ICAO. This methodology is derived from US Environmental Protection Agency (EPA), which is commonly used by many researchers. The calculation formula is presented as follows:

$$\begin{array}{l} E_{i}=\underset{j}{\sum }\overset{4}{\underset{m=1}{\sum }}s_{j}\times n_{j}\times F_{j,m}\times t_{j,m}\times {EI}_{i,j,m} \end{array}$$

Where

*i*: the type of the emission (i.e., HC, NO_x_, and CO)

*j*: the type of the aircraft

*m*: the operation mode in the LTO cycle (e.g., idle, approach, climb-out, and take-off)

*E*_*i*_: Total emissions of pollutant *i* in specific period, in grams (g)

*s*_*j*_: the total number of the LTO cycle for aircraft type *j*

*n*_*j*_: the number of engines used on aircraft type *j*

*F*_*j,m*_: the fuel flow for mode *m* for each engine used on aircraft type *j*, in kilograms per second (kg/s)

*t*_*j,m*_: Time in mode for mode *m*, in second

*EI*_*i,j,m*_: the emission factor for pollutant *i*, mode *m*, in grams per pollutant per kilogram of fuel (g/kg of fuel) for each engine used on aircraft type *j*.

#### Spatial-Temporal Analysis

Using values from the calculation model, we investigated the variation pattern of the emission quantity in the research region on a monthly base from January to December in year 2020. Considering different capacities of the airports, 22 airports in this region are divided into several groups for comparison. After the temporal analysis of the monthly variation, the whole year of 2020 was further divided into 3 phases for the spatial distribution analysis. Each phase consists of 4 months. Phase I is from January to April. Phase II is from May to August. Phase III is from September to December. The purpose of spatial distribution analysis is to investigate how the emission quantities of HC, CO, and NO_x_ are arranged and distributed across airports with different geographical locations in the Yangtze River Delta. Using ArcGIS, the distribution pattern of the three air pollutants emissions is displayed on the maps. Different colors on the maps represent the imparities of emissions in the region. By comparing those maps, the distribution pattern of the three air pollutants is illustrated. The impact of COVID-19 on the variation and distribution pattern will also be discussed.

## Results

### Time Series Analysis

#### Shanghai

Being a global center for finance, manufacturing, technology and many other domains, Shanghai is one of the busiest aviation hubs in Asia-Pacific region. Currently, the city has two commercial airports: PVG and SHA. As for all the flights departing and arriving in Shanghai, PVG handles 60% of them while the remaining 40% use SHA. However, being adversely influenced by COVID-19, the ranking of PVG by passenger volume fell behind SHA for the first time in years due to the plummet of international flights. Because of the huge throughput of PVG and SHA, the quantities of air pollutants emissions are also far more than the other airports in the Yangtze River Delta.

**Figure 2:**
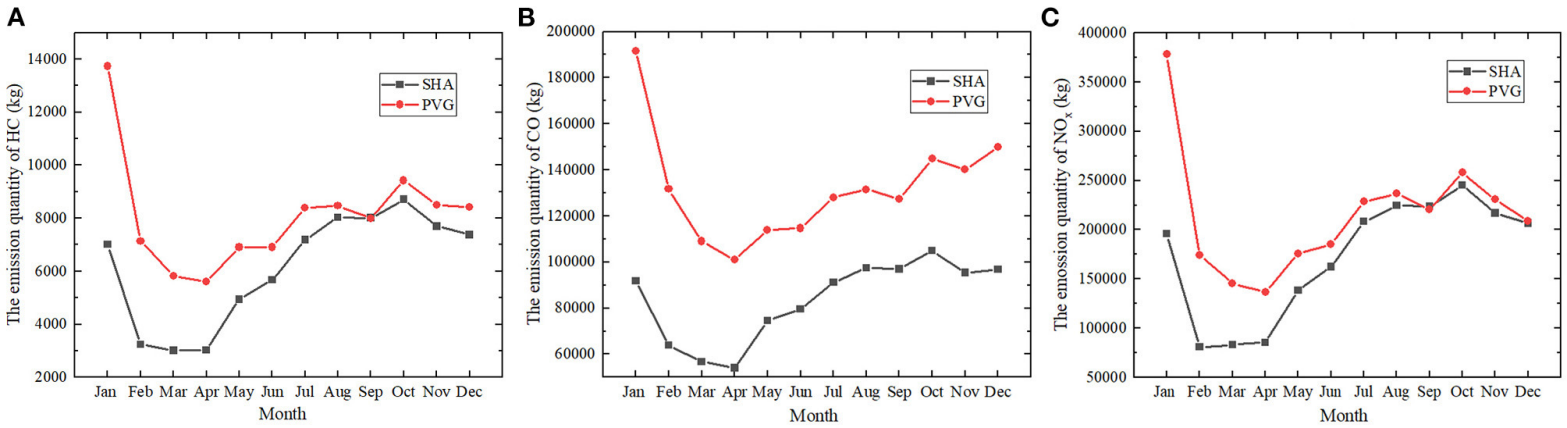
The monthly variation of the air pollutants at 2 airports (i.e., PVG amd SHA) in Shanghai, 2020. **(A)** The emission quantity of HC in kilograms **(B)** The emission quantity of CO in kilograms **(C)** The emission quantity of NO_x_ in kilograms. PVG, Shanghai Pudong Airport; SHA, Shanghai Hongqiao Airport.

The first confirmed case of COVID-19 in Shanghai was identified on 20 January 2020. Two days after, the government of Shanghai started the initiation of Level 1 Response for major public health emergency. From 10 February, the person who is not resident in Shangai was banned from entering the city. From 4 March, passengers who had been in Korea, Japan, Italy, and some other countries would have to quarantine themselves for 14 days. This policy was subsequently further expanded to 8 countries 8 days after. All those measures led to the downturn in flights. As can be seen in Figure 2, the emission quantities of HC, CO, and NO_x_ all decreased dramatically in February at two airports in Shanghai. The reductions, up to ~51% for HC, 30% for CO, and 56% for NOx, are statistically significant compared to the same period in 2019. The level of studied aircraft pollutants at PVG further declined to the bottom in April, at around 5,605 kg for HC, 101,000 kg for CO, and 137,000 kg for NOx, respectively. In contrast, the emission quantity of NO_x_ at SHA slightly increased by 2.94% in March and 2.78% in April. There were continual upsurges in the three emissions during the next four-month period. But the conditions dropped slightly from October to the end of 2020 except for the emission of CO at PVG, which fell from around 145,000 to 140,000 kg before increasing at 150,000 kg in December.

#### Jiangsu Province

With its capital city in Nanjing, Jiangsu is the third smallest but the most densely populated province in China. It is also the province which has the largest number of airports: NKG, CZX, HIA, LYG, NTG, WUX, XUZ, YNZ, and YTY. Located within the capital city, NKG ranked 11 in 2019 with approximately 235,000 aircraft movements. This number dropped to 182,000 in 2020. The second busiest airport in Jiangsu is WUX which has more than 53,000 flights in 2020. That figures for the other airports are from 20,000 to 40,000 with the outlier of LYG, which only had 12,000 aircraft movements.

**Figure 3:**
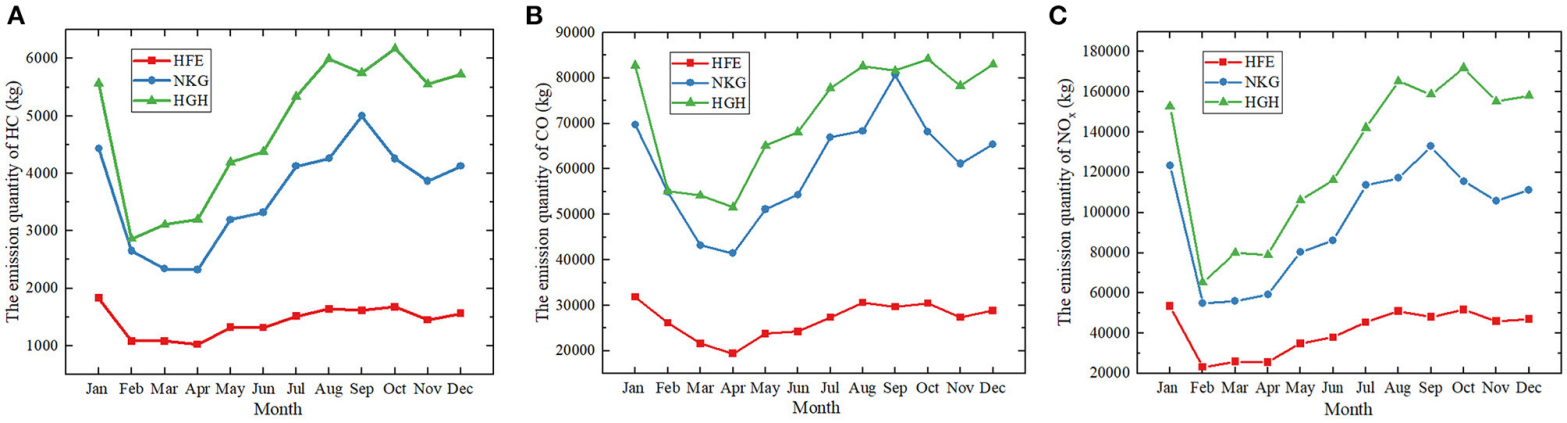
The monthly variation of the air pollutants at airports in the 3 capital cities (i.e., Nanjing, Hangzhou, Hefei), 2020. **(A)** The emission quantity of HC in kilograms **(B)** The emission quantity of CO in kilograms **(C)** The emission quantity of NO_x_ in kilograms. HFE, Hefei Xinqiao Airport; NKG, Nanjing Lukou Airport; HGH, Hangzhou Xiaoshan Airport.

**Figure 4:**
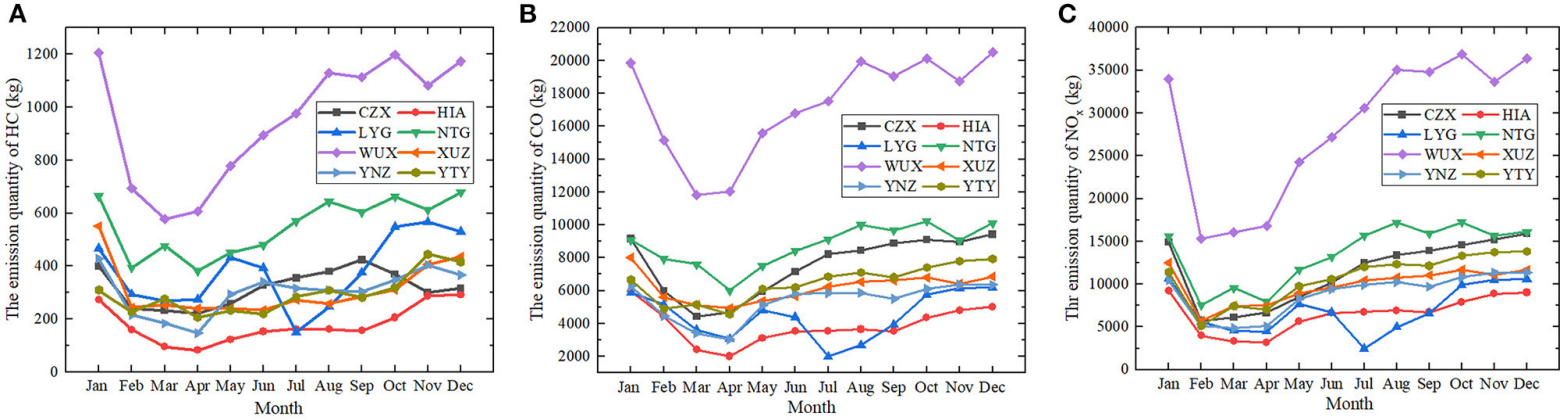
The monthly variation of the air pollutants at 8 airports in Jiangsu province (except for NKG), 2020. **(A)** The emission quantity of HC in kilograms **(B)** The emission quantity of CO in kilograms **(C)** The emission quantity of NO_x_ in kilograms. CZX, Changzhou Benniu Airport; HIA, Huaian Lianshui Airport; LYG, Lianyungang Baitabu Airport; NTG, Nantong Xingdong Airport; WUX, Sunan Shuofang Airport; XUZ, Xuzhou Guanyin Airport; YNZ, Yancheng Nanyang Airport; YTY, Yangzhou Taizhou Airport.

Under the context of COVID-19 in 2020, the monthly average emissions of HC, CO, and NO_x_ emitted at airports in Jiangsu province decreased by 24.3, 11.3, and 18.2% compared to those in 2019. In terms of all the three air pollutants, sharp declines were observed in February, with average percentage decrease of around 41.5% for HC, 23.9% for CO, and 54.3% for NO_x_. Such reduction was in expectation considering the fact that the first confirmed case of COVID-19 was found on 23 January in Jiangsu province. As for the emission of HC, the quantity at NTG and YTY went up slightly by 20.8 and 22.4% in March and then dropped again by 19.7 and 25.9% in April. While the patterns for WUX had changed in a contradictory way. The emission quantities fell to the bottom at ~577 and 269 kg and then increased by 5.2 and 2.2%, respectively. The conditions for the other airports all fell gradually in those 2 months. The variation patterns of CO and NOx are comparable to the variation pattern of HC, with minor differences at some airports. Despite some fluctuations, the emissions of all three pollutants at the airports in Jiangsu province rebounded back to the level of January at the end of 2020. The conditions of the pollutants at airports like WUX, NTG and YGY even showed elevated levels in January, indicating the recovery of civil aviation from the ravages of the pandemic in these districts (Figures 3, 4).

#### Zhejiang Province

With its capital city in Hangzhou, Zhejiang is considered as one of the wealthiest provinces in China, ranking fourth in GDP nationally as of 2019 and 2020. There are currently 6 commercial airports in Zhejiang province: HGH, NGB, HYN, WNZ, YIW, and HSN. Handled more than 237,000 flights in 2020, HGH in Hangzhou city is the 10th busiest airports at the national scale. It is also a hub for several major airlines in China, including Air China, China Eastern Airlines, China Southern Airlines, etc. The second and third busiest airports in Zhejiang is NGB and WNZ, with ~74,000 to 75,000 aircrafts movements respectively in 2020. That number in HYN is less than 10,000.

**Figure 5:**
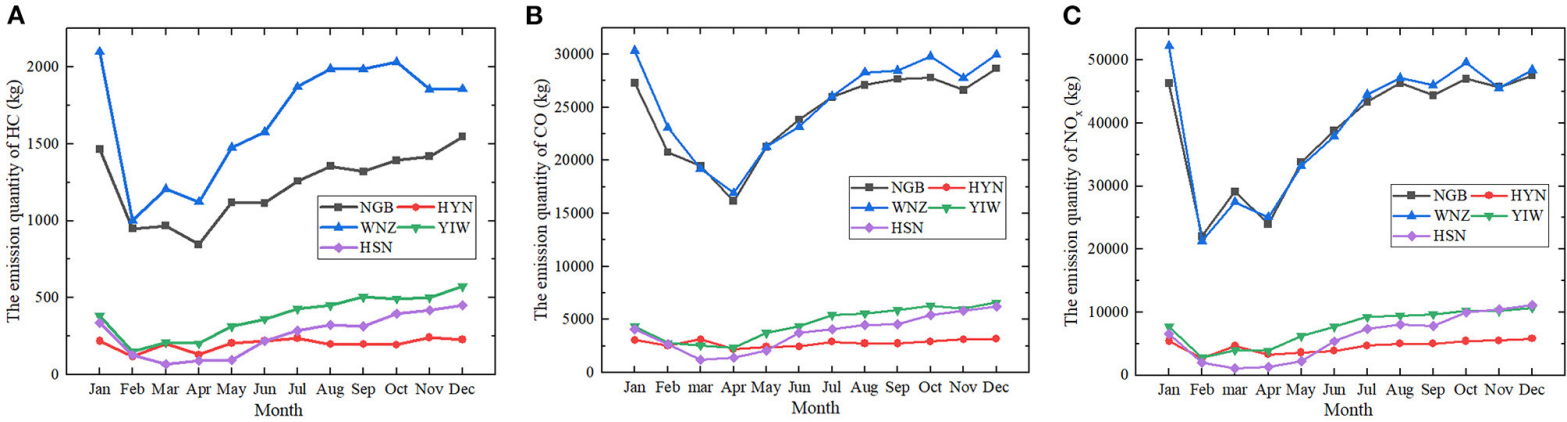
The monthly variation of the air pollutants at 5 airports (except for HGH) in Zhejiang province, 2020. **(A)** The emission quantity of HC in kilograms **(B)** The emission quantity of CO in kilograms **(C)** The emission quantity of NO_x_ in kilograms. NGB, Ningbo Lishe Airport; HYN, Taizhou Luqiao Airport; WNZ, Wenzhou Longwan Airport; YIW, Yiwu Airport; HSN, Zhoushan Putuoshan Airport.

Due to the high frequency of travel with Wuhan, Zhejiang was the third worst-affected province in February 2020. It is also the first province to announce the highest level of public health emergency in response to the pandemic on 23 January 2020 (14). Accordingly, a drastic fall was particularly noticeable for all pollutants of the study scope at all airports in February. Compared to the first month of 2020, the average decrease of HC, CO, and NO_x_ was 50.9, 28.2, and 58.5%, respectively. The next 2-months period witnessed quite different variation patterns of three emissions at airports in Zhejiang. In terms of CO, the emission quantities at all airports except HYN continued to decline until reaching the lowest points in April. In contrast, the emission quantities of HC and NO_x_ experienced obvious fluctuations during this period. During the four-month period following January, the emissions of HC, CO, and NO_x_ at airports in Zhejiang province, except HGH reduced by 9.9, 9.1, and 11.4% compared to the same period in 2019. After the end of April, the emissions at most of the airports increased gradually and reached the peak in December. However, the emissions at HGH and WNZ eventually decreased slightly in the last 2 months of the year 2020 (Figures 3, 5).

#### Anhui Province

With its capital city in Hefei, Anhui province is the only landlocked province in Yangtze River Delta. There are 5 major commercial airports in Anhui: HFE, TXN, FUG, AQG, and JUH. The civil aviation industry of Anhui province is much less developed than Jiangsu and Zhejiang province. As the airport in the capital city, HFE ranked 34 at national scale by passenger volume in 2020, one place behind WNZ. It reported around 74,000 flights in 2020, which is much less than the other 2 capital cities in the study region. Apart from FUG, which handles nearly 14,000 flights, the numbers of the aircraft movements in AQG, JUH and TXN were all below 6,000 in 2020.

**Figure 6:**
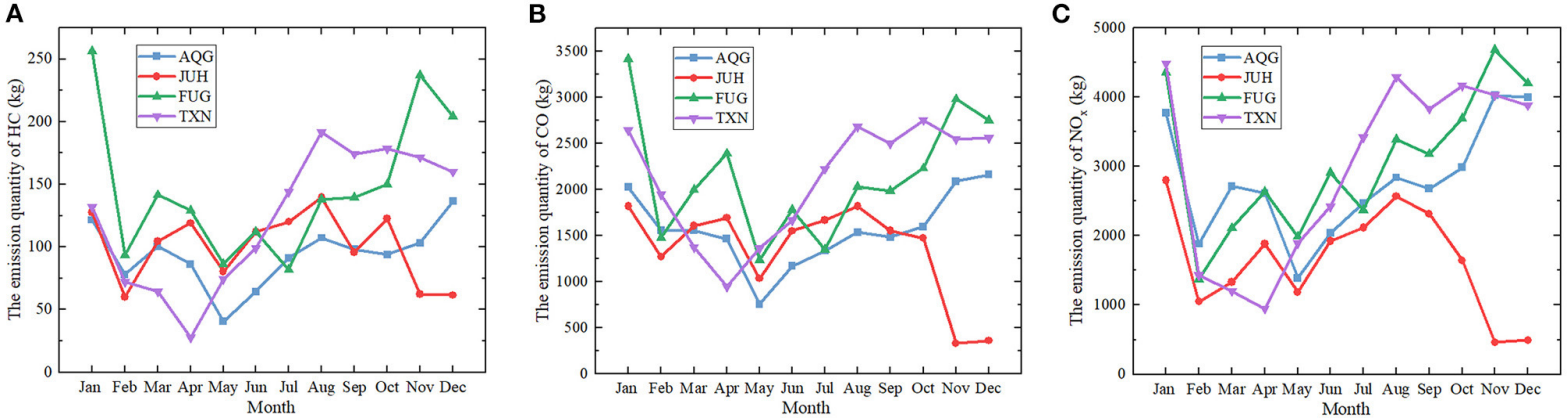
The monthly variation of the air pollutants at 4 airports (except for HFE) in Anhui province, 2020. **(A)** The emission quantity of HC in kilograms **(B)** The emission quantity of CO in kilograms **(C)** The emission quantity of NO_x_ in kilograms. AQG, Anqing Tianzhushan Airport; JUH, Chizhou Jiuhuashan Airport; FUG, Fuyang Xiguan Airport; TXN, Huangshan Tunxi Airport.

Unlike Jiangsu and Zhejiang province, the emission quantities at airports in Anhui province experienced more obvious fluctuations over the whole period in 2020. Compared to 2019, there was a 16.9% reduction in HC, a 11.3% reduction in CO, and a 18.2% reduction in NO_x_. At HFE, the emission quantities of HC and CO decreased from January to April by 44.1 and 39% before rallying to the peak at 1,641 and 30,651 kg in August, whilst the quantity of NO_x_ slightly increased by about 11.4% in March compared to February. All the emissions leveled off from August to the end of 2020 at HFE, with ~1,588 kg for HC, 29,396 kg for CO, and 48,777 kg for NO_x_, respectively. In terms of TXN, the emissions of the pollutants in this study showed a downward trend during the first season of 2020, with a decline of 79% for HC, 64.3% for CO, and 78.9% for NO_x_. These conditions surged from April to August and dwindled in the last 4 months. In regard to the other three airports in Anhui, the first 10 months witnessed unstable rises and falls of the emissions. After October, the emissions at JUH declined again while the conditions of TXN changed in an antithetical manner, which greatly widened the contrast with JUH (Figures 3, 6).

### Spatial Pattern Analysis

**Figure 7:**
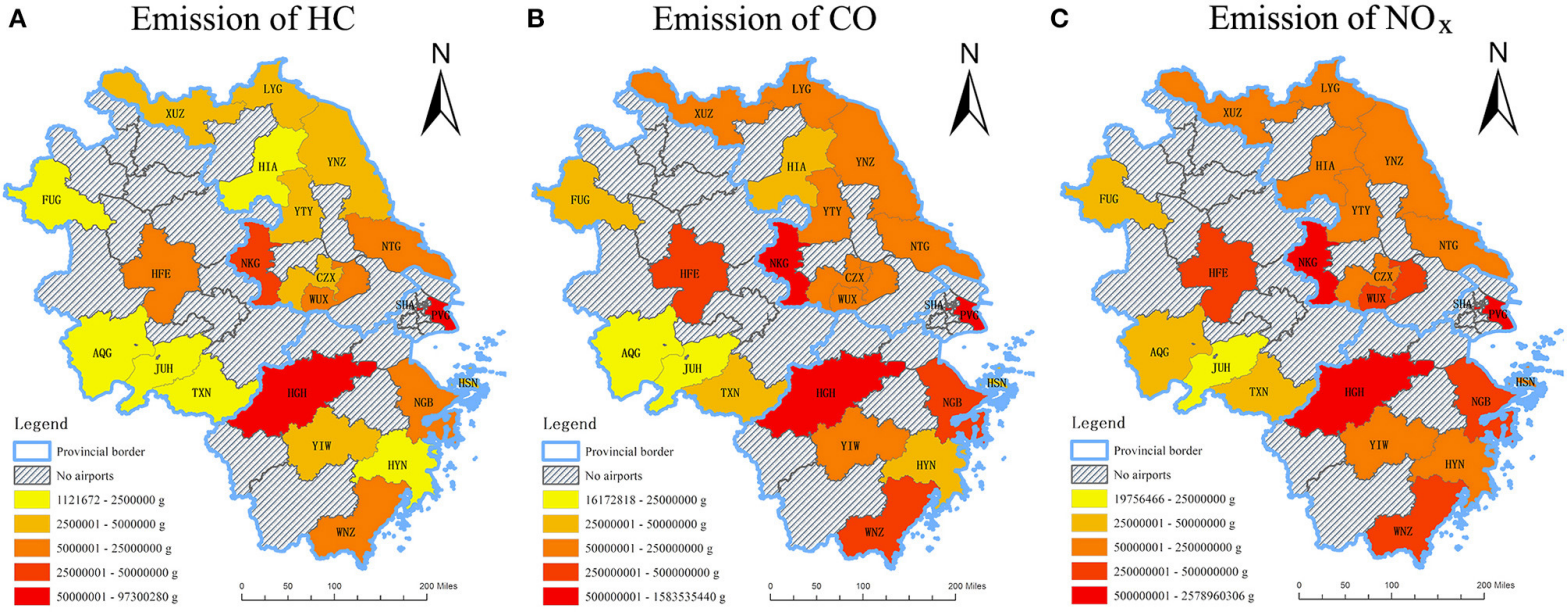
Total emission quantities of HC, CO, and NO_x_ in spatial scale, 2020. **(A)** Emission quantity of HC **(B)** Emission quantity of CO **(C)** Emission quantity of NO_x_.

At airports in the Yangtze River Delta, the relative change between historical monthly average emissions of HC, CO, and NO_x_ in 2019 and those during the outbreak of COVID-19 in 2020 ranged from −47.7 to −2.9% for HC, −35.8 to −0.8% for CO, and −39.6 to −1.6% for NO_x_, respectively. As can be seen in Figure 7, for entire year in 2020, the quantities of NO_x_ emitted at each airport are far more than the quantities of HC and CO, indicating that NO_x_ is the dominant emissions among the three gaseous pollutants. In terms of spatial distribution, airports in Shanghai released the largest quantities of emissions for each of the three air pollutants, with PVG ranked first and SHA ranked the second among all 22 airports. The emissions at airports in Shanghai constituted about 45.1% of total HC, 42.9% of total CO, and 45.2% of total NO_x_ in the Yangtze River Delta. This phenomenon is consistent with the fact that Shanghai is the aviation hub for both domestic and international travelers. Among the three provinces of the research region, Zhejiang Province took up the largest emission proportion of HC (49.2%), CO (46.3%), and NO_x_ (48.1%) respectively, despite having 3 fewer airports than Jiangsu Province. Anhui Province has the least emissions from LTO cycles at airports, the number of which is about one-seventh of that figure for Shanghai city. Furthermore, distinct differences can be found between airports in each province. Evidently, airports located in the capital cities of three provinces (i.e., Nanjing, Hangzhou, and Hefei) always produced the most emissions and the quantities of which were usually much higher than those figures of the rest of the cities within the province.

In general, the spatial distribution of the emission quantity declined from the east coast to the central and western part of the Yangtze River Delta for all the three phases of 2020. Among three capital cities (i.e., Nanjing, Hangzhou, and Hefei), significant reductions of three studied air pollutants from LTO cycles only occurred in NKG and HGH where NO_x_ declined the most, while the changes in the aircraft emissions at HFE were relatively minor. The emission quantity of airports within Shanghai and the three capital cities of the provinces were always beyond the provincial average, which is correlated to the fact that these airports hold the dominant market positions of civil aviation in this region. As for the other airports, data sources that are geographically closer to Shanghai released more emissions for all periods.

**Figure 8:**
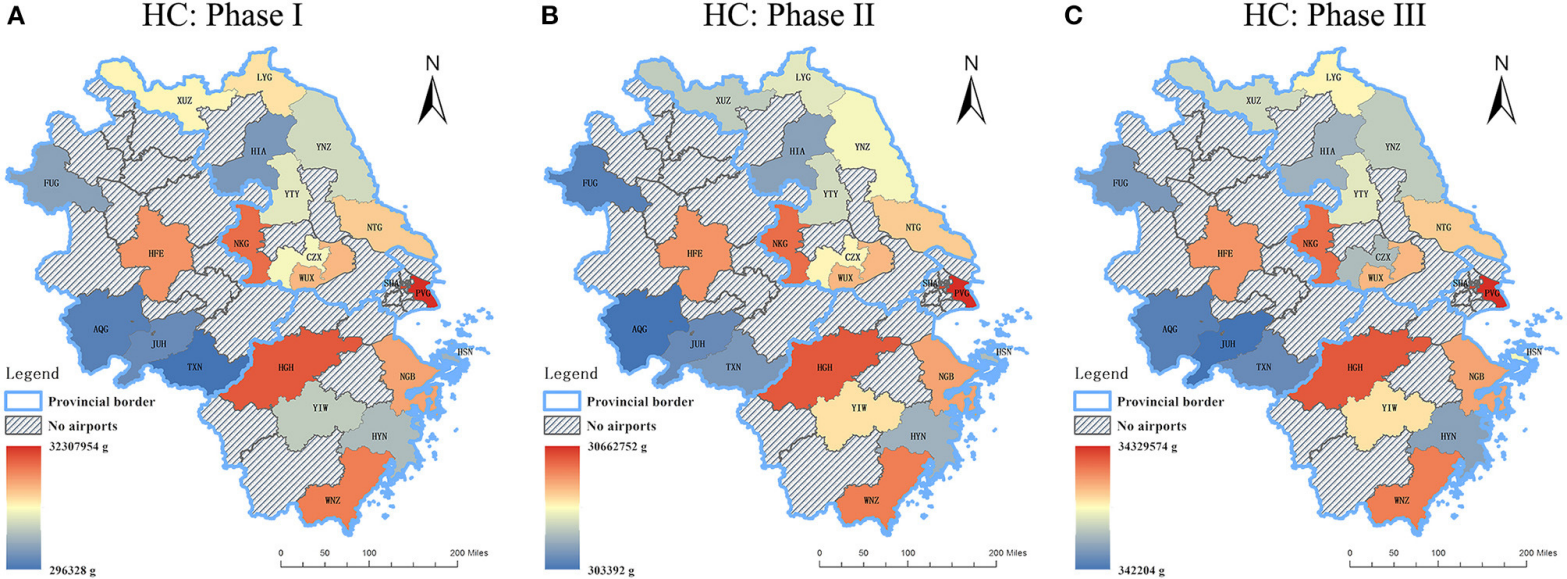
The emission quantity of HC during the three phases of 2020. **(A)** Phase I : from the beginning of January to the end of April **(B)** Phase II: from the beginning of May to the end of August **(C)** Phase III: from the beginning of September to the end of December.

**Figure 9:**
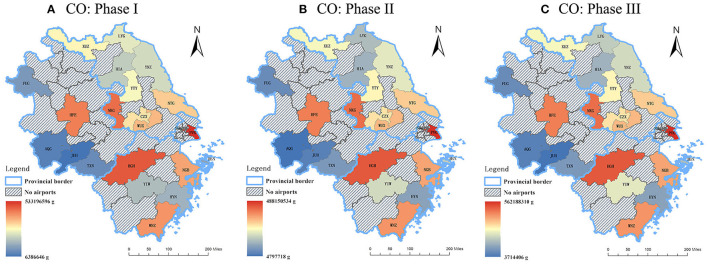
The emission quantity of CO during the three phases of 2020. **(A)** Phase I: from the beginning of January to the end of April **(B)** Phase II: from the beginning of May to the end of August **(C)** Phase III: from the beginning of September to the end of December.

**Figure 10:**
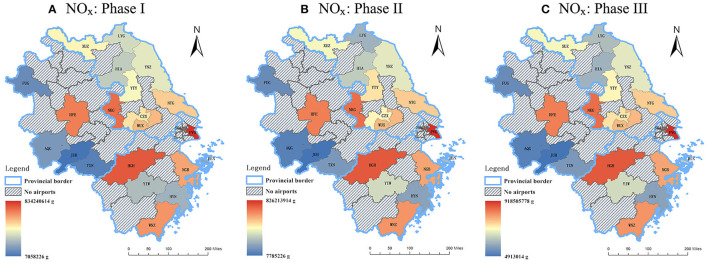
The emission quantity of NO_x_ during the three phases of 2020. **(A)** Phase I: from the beginning of January to the end of April **(B)** Phase II: from the beginning of May to the end of August **(C)** Phase III: from the beginning of September to the end of December.

From Figures 8–10, the following results can be found. Compared to Phase I, the emission quantities at most of the airports in the Yangtze River Delta in Phase II increased at different rates. Among all the airports, YIW which is located within Yiwu city of Zhejiang Province had the most significant increases of HC, CO, and NO_x_, which were 64.32, 58.71, and 76.40%, respectively. Heralding a strong domestic and international trade network, Yiwu is widely known as the international trade city. As one of the barometers for the health of the nation's exports, Yiwu suffered from the impacts of COVID-19 on exportation during the first quarter of the year. However, after the pandemic being controlled in China after March, production line in Yiwu gradually resumed. Sizable orders from clients of different domains aided in financially bolstering the air freight industry, which may be attributed to the obvious growth of emissions in Phase II. In terms of the airports in Shanghai, the emission quantity at PVG dropped in Phase II, with reductions of 5.09% in HC, 8.45% in CO, and 0.96% in NO_x_, respectively. Conversely, the patterns of emissions at SHA showed an upward trend, with increases of 58.53% in HC, 28.64% in CO, and 64.74% in NO_x_, respectively. Such phenomenon can be explained by the different functional positions of these two airports in Shanghai. SHA is the main hub for most of the national flights, whilst PVG fills a considerable international demand. After April, the pandemic in other parts of the world becomes more and more serious. On March 12, 2020, Civil Aviation Administration of China (CAAC) issued the Five International Flight Plans (Phase Five), which limited the number of international routes and inbound flights from other countries. This policy was further adjusted in June. The governmental restriction on international flights serves as an explanation as to why the emission quantity at PVG decreased in Phase II.

Compared to Phase II, the emission quantities at all airports except JUH enjoyed marked growth at different increase percentages in Phase III. As for HC, Jiangsu province had the highest average increase, which was 31.11%. As for CO and NO_x_, Zhejiang province had the highest average increases, which were 22.96 and 20.19%, respectively. Among all the airports, the total emission quantity at LYG, HSN, AQG and FUG experienced the most significant rises in Phase III, with an increase of 67, 64.1, 55, and 51.3%, respectively. It should be noted that these cities are akin in the manner that these airports have a larger portion of tourist traffic as they are located in cities with close proximity to tourist attractions. Given the fact that there are several public holidays in Phase III, such as Chinese National Day, Mid-Autumn Festival, etc., it is understandable to observe the rise in flights which led to the spike in emission quantities from LTO cycles in those cities. The airports (NKG, HGH, and HFE) located in three capital cities experienced steady increase at moderate rates in the emission quantity.

## Discussion

Previous studies have shown that significant reduction in air pollution was found in China (15–17), Italy (18, 19), the U.S. (20–22), Japan (23, 24), and many other nations in the world due to travel restrictions during the pandemic (6). Recent data from National Aeronautics and Space Administration (NASA) and European Space Agency (ESA) shows that pollution in China, Italy, the U.S., and some other epicenters of COVID-19 has reduced up to 30% (25). Tobías et al. (26) found that NO_2_ and BC decreased by 50% during the lockdown. Wang and Su (27) found that full or partial lockdown had also led to substantial reduction in greenhouse gases in China. Dantas et al. (28) pointed out that both NO_2_ and PM_10_ reduced to a low level in a region of Brazil. However, those results are mostly based on the air quality monitoring stations, which traces the trends of criteria air pollutants (CAPs) in a particular region. Therefore, when it comes to a specific transportation industry, e.g., civil aviation, it is difficult to monitor the accurate amounts of CAPs which a specific industry generates over a period.

There are also a few relevant studies focusing on the measurement of Aircraft Pollutant Emissions (APEs) during the LTO cycles. Organizations like ICAO and EPA have their own standards for the calculation of the APEs. Yilmaz (29) presents the estimation for APEs at Kayseri Airport in 2010. Song and Shon (30) estimated the greenhouse gases in multiple airports in South Korea over a 2-year time span. Hu et al. (13) analyze the variation of APEs during the LTO phase in Jiangsu Province of China from 2007 to 2016. Wasiuk et al. (31) developed the Aircraft Performance Model Implementation (APMI) software to study global aircraft emissions. These studies made thorough analysis on the variation pattern of APEs over different time spans at the airport level, regional level and global level. However, the research time of these studies all occur prior to 2020 whilst the aviation industry was operating under pre-virus conditions. Given that 2020 is a uniquely challenging year due to a severe global health threat that catastrophically hit the aviation industry, it is of necessity to study the impact of such pandemic on the aviation environment.

The study in this paper filled the gaps mentioned above. It is the first study that assesses the temporal and spatial variation pattern of HC, CO, and NO_x_ at airports in the Yangtze River Delta under the context of COVID-19 pandemic. It is also an initial effort to understand the correlation between the COVID-19 responses and the aircraft emissions from the LTO cycles. However, the impact of the measurements and policies on the reduction of the aircraft emissions is only qualified in this paper. Given the possibility that the variation of the emission quantity can also be resulted from some other factors, such as the climate change and global warming, further studies that quantitatively consider the correlation between the epidemic and emissions are of necessity.

Apart from the work and contribution made by this research, there are further works that can be done in the future to better understand this subject, including:

(1) Including the comparison between 2020 and the previous years when COVID-19 pandemic had not yet occurred regarding the aircraft emission from the LTO cycle.

(2) Conducting similar researches in other parts of China, especially the epicenters of the COVID-19 outbreak, and make comparison between those results.

(3) Investigating the impacts of other socio-economic factors (e.g., GPD per capita, population) on the variation patterns of the emissions at different airports in the Yangtze River Delta.

## Conclusions

According to the calculation and spatial-temporal analysis, this study has come to the following conclusions:

(1) Although the COVID-19 pandemic has caused a collapse in civil aviation industry, gaseous pollutants like HC, CO, and NO_x_ at airports have also decreased due to the travel restrictions and less desire by the passengers to travel.

(2) The air pollutant emissions at ground level are determined by the number of LTO cycles, which is relevant to the number of aircraft movement, as well as the aircraft type, thrust setting and typical time spent in each mode of operation. The emission quantity shows a strong relationship with the capacity of each airport, thus demonstrating that the airports which handle more flights in a given year generally release more emissions.

(3) The quantity of NO_x_ in each airport is far more than the quantities of HC and CO, indicating that NO_x_ is the dominant emission among the three gaseous pollutants.

(4) The variation pattern of the three air pollutants (i.e., HC, CO, and NO_x_) from LTO cycles were significantly influenced by the COVID-19 pandemic. During the outbreak of the epidemic in February, the emission quantity of all three air pollutants experienced sharp decline at all the airports in the Yangtze river delta. Despite some fluctuations in the middle of the year, the emission quantity at most of the airports rebounded to the levels observed before the outbreak of COVID-19 at the end of 2020.

(5) The spatial distribution analysis showed that there exists specific patterns of the emission quantity of the three air pollutants at airports in the research region. Generally, the emission quantity declined from the east coast to the central and western part of the Yangtze River Delta. Additionally, airports that are geographically closer to Shanghai released more emissions.

(6) Discrepancies in the target markets create disparities in the variation pattern of emission quantities at different airports. Airports that serve primarily to domestic markets tended to recover faster than airports that mostly serve international flights. Airports that are located in the city which strongly rely on tourism have witnessed substantial growth in the emission quantity during Phase III.

## Data Availability Statement

The raw data supporting the conclusions of this article will be made available by the authors, without undue reservation.

## Author Contributions

DB: conceptualization, formal analysis, methodology, and writing—original draft. ST: formal analysis, methodology, and writing—original draft. ZZ: data collection and data calculation. HC: formulation of figures. TZ and NC: review and editing. All authors contributed to the article and approved the submitted version.

## Conflict of Interest

The authors declare that the research was conducted in the absence of any commercial or financial relationships that could be construed as a potential conflict of interest.

## Publisher's Note

All claims expressed in this article are solely those of the authors and do not necessarily represent those of their affiliated organizations, or those of the publisher, the editors and the reviewers. Any product that may be evaluated in this article, or claim that may be made by its manufacturer, is not guaranteed or endorsed by the publisher.
